# Exploring the Y Chromosomal Ancestry of Modern Panamanians

**DOI:** 10.1371/journal.pone.0144223

**Published:** 2015-12-04

**Authors:** Viola Grugni, Vincenza Battaglia, Ugo Alessandro Perego, Alessandro Raveane, Hovirag Lancioni, Anna Olivieri, Luca Ferretti, Scott R. Woodward, Juan Miguel Pascale, Richard Cooke, Natalie Myres, Jorge Motta, Antonio Torroni, Alessandro Achilli, Ornella Semino

**Affiliations:** 1 Department of Biology and Biotechnology, University of Pavia, Pavia, Italy; 2 Sorenson Molecular Genealogy Foundation, Salt Lake City, Utah, United States of America; 3 Department of Chemistry, Biology and Biotechnology, University of Perugia, Perugia, Italy; 4 Instituto Conmemorativo Gorgas de Estudios de la Salud, Panama City, Panama; 5 Smithsonian Tropical Research Institute, Panama City, Panama; 6 Ancestry, Provo, Utah, United States of America; Universitat Pompeu Fabra, SPAIN

## Abstract

Geologically, Panama belongs to the Central American land-bridge between North and South America crossed by *Homo sapiens* >14 ka ago. Archaeologically, it belongs to a wider Isthmo-Colombian Area. Today, seven indigenous ethnic groups account for 12.3% of Panama’s population. Five speak Chibchan languages and are characterized by low genetic diversity and a high level of differentiation. In addition, no evidence of differential structuring between maternally and paternally inherited genes has been reported in isthmian Chibchan cultural groups. Recent data have shown that 83% of the Panamanian general population harbour mitochondrial DNAs (mtDNAs) of Native American ancestry. Considering differential male/female mortality at European contact and multiple degrees of geographical and genetic isolation over the subsequent five centuries, the Y-chromosome Native American component is expected to vary across different geographic regions and communities in Panama. To address this issue, we investigated Y-chromosome variation in 408 modern males from the nine provinces of Panama and one indigenous territory (the comarca of Kuna Yala). In contrast to mtDNA data, the Y-chromosome Native American component (haplogroup Q) exceeds 50% only in three populations facing the Caribbean Sea: the comarca of Kuna Yala and Bocas del Toro province where Chibchan languages are spoken by the majority, and the province of Colón where many Kuna and people of mixed indigenous-African-and-European descent live. Elsewhere the Old World component is dominant and mostly represented by western Eurasian haplogroups, which signal the strong male genetic impact of invaders. Sub-Saharan African input accounts for 5.9% of male haplotypes. This reflects the consequences of the colonial Atlantic slave trade and more recent influxes of West Indians of African heritage. Overall, our findings reveal a local evolution of the male Native American ancestral gene pool, and a strong but geographically differentiated unidirectional sex bias in the formation of local modern Panamanian populations.

## Introduction

Panama is the southernmost country of Central America delimited by Costa Rica to the west, Colombia to the south-east, the Caribbean Sea to the north and the Pacific Ocean to the south. Geologically, Panama is part of a natural land-bridge between the two continental American landmasses. Archaeologists include it in a wider Isthmo-Colombian Area [1]. The earliest well documented cultural remains in Panama refer to the Clovis tradition dated to ~11,000 years BP (13,200 calibrated 14C years) [2]. However, mtDNA data [3] infer a sizeable human presence before the Clovis along the Pacific littoral of Panama and Costa Rica. Pre-Clovis archaeological sites are likely to now lie submerged on the Pacific continental shelf of Panama and be similar in culture and customs to pre-Clovis sites in Colombia and Venezuela, e.g., Tibitó and Taima-Taima, where extinct megafauna was exploited [4].

Archaeological and paleoecological data concur that some descendants of the initial colonizing populations remained in Panama and Colombia while others moved southward [5–7]. They adapted their way of life to changing environmental conditions resulting from the continental processes of the Late Glacial-Holocene transition. They set in motion anthropogenic effects on the landscape that are most pronounced in areas with seasonal climates. Cultural and population continuity after the Clovis horizon and until the onset of the Holocene is attested by some lake sediments and archaeological areas [5].

### Agriculture emergence and cultural diversity

Agriculture prospered across the Isthmo-Colombian Area including Panama after ~8,000 years BP using an increasing number of cultivars [7–9]. Although most cultivars, such as maize (*Zea mays*), squash (*Cucurbita* spp), and manioc (or cassava) (*Manihot esculenta*), were first domesticated north or south of the Panama land-bridge, there is no evidence for their introduction having been accompanied by a wave-like spread of agriculture and whole populations as hypothesised in Eurasia [10, 11]. Rather, it is inferred that seeds and cuttings and the few domesticated animals, like muscovy duck (*Cairina moschata*), moved through a multi-directional network of small farming communities including by sea in and around the Caribbean [6, 7, 12].

Nuclear Chibchan languages are the most widely spoken linguistic group in Panama today (S1 File). Human population geneticists and archaeologists have attributed its multiple branching to increasing sedentism, and the resulting spatial fragmentation of descendant populations within the Isthmo-Colombian Area [13–16].

After ~4,000 years BP, villages grew rapidly in number and size especially in areas characterized by fertile soils and abundant fluvio-estuarine resources [17]. Gradually, villages were incorporated into larger territories generally described as “chiefdoms”, and multi-tiered regional hierarchies formed. Although kin-based elites increasingly acquired prestige goods from afar, most trade was between nearest neighbours taking advantage of resource diversity afforded by lower isthmian micro-geography and micro-climate [17–19]. Further vertical and horizontal linguistic diversification ensued among the Nuclear Chibchan languages: the Ngäbe, Buglé and Kuna languages spoken in areas that provided Y-chromosome data for this project, are inferred to have coalesced as an eastern isthmian clade of the isthmic lineage about 5,000 years BP [16].

### Isthmian and extra-isthmian contacts before Spanish conquest

During the last five centuries of the pre-Columbian era, increasing evidence for connections between eastern Panama and the lowlands of northern Colombia infer cultural contacts that went beyond the exchange of goods and ideology and may have involved displacements of people. Even so, these appear to have involved only short-distance movements into eastern Panama from other nearby regions of the Isthmo-Colombian Area. Ridged fields, similar to much more extensive field systems in the San Jorge floodplain in the Atlantic lowlands of Colombia, were recently observed at Chinina in Panamá province. The post-1,000 years BP pottery recovered here belongs to a tradition called “Gran Darién” that is widespread in Panamá province, Colón, Darién and Kuna Yala [20]. This pottery tradition is coterminous with the territory occupied at Spanish contact by a culturally heterogeneous people who used the “language of Cueva” [14, 21, 22] arguably not as a vernacular, but rather as a language of communication in a polyglot society [14].

### Spanish conquest and Native American survival in Panama

A drastic change occurred on the Central American land-bridge and in northernmost Colombia during the first three decades of Spanish incursions (1499–1530). It is well-known that these, in conjunction with diseases that rampaged through the entire indigenous population lacking immunity, caused a huge impact on the large indigenous populations, and their socio-cultural and economic systems. These repercussions, however, show a strong geographic bias, being strongest in areas where the millennial anthropogenic landscapes, preferred by the Spanish, dominated the pre-conquest landscape. Depopulation quickly ensued, accentuated by the multiple effects of unequal combat among males and the selection of healthy males for work in the gold mines. Mortality among the Native American population clearly had a sex bias with more men perishing or being deprived of sexual rights than women. Many women passed quickly into the Hispanic sphere transmitting their genes to the post-colonial population [23, 24].

The first authorized importation of black African slaves dates to 1523 [25]. Eventually, more than 5,000 sub-Saharan African slaves were employed in the transit zone between Panama City and the Caribbean port of Nombre de Dios (Colón), and up to 2,000 in gold mines in central Caribbean Veraguas. After emancipation, small communities of Spanish-speaking black ex-slaves grew up around Spanish rural towns. Starting in the early 19^th^ century, small numbers of Afro-descendants from the English-speaking West Indian islands and the Caribbean islands settled along the western Caribbean coast, mostly in Bocas del Toro. There, they entered into contact with Native American communities [26]. Between 1850 and 1914, other people of sub-Saharan descent, mostly from Caribbean islands, concentrated in the transit zone during the construction of the trans-isthmian railroad and the French and US canals [27].

The present-day population of Panama is mainly concentrated in the urbanized corridor between Panama City and Colón. Three areas, termed “Comarcas”, with a substantial presence of indigenous population exist in Panama: Comarca Emberá-Wounaan, Kuna Yala, Ngäbe-Buglé. They are administrative regions equivalent to provinces. In 2010 Panama comprised more than 3,600,000 inhabitants (http://www.contraloria.gob.pa/inec/) and was quite heterogeneous: about 65% was represented by “Mestizos” (i.e., a tri-hybrid population of descendants of Europeans, sub-Saharan Africans and Native Americans); 16% by sub-Saharan Africans; 7% by Europeans, and only 12% (417,559) by Native Americans. The most numerous Native American cultural groups are the Ngäbe (62.3%), Kuna (19.3%) and Emberá (7.5%) followed by smaller cultural groups: the Wounáan, Bribri, and Naso Djërdi (or Teribe). The Ngäbe, Buglé, Naso Djërdi, Bribri and Kuna speak languages in the isthmian lineage of the Nuclear Chibchan languages [16, 28]. The Emberá and Wounaan speak dialects of two languages of another less diffused language family called Chocoan [22, 29]. While some scholars argue that ancient forms of these languages were spoken in eastern Panama [14, 21, 22], others claim that Chocoan populations moved from north-west Colombia, e.g. the Baudo, Cauca, San Juan and Atrato rivers [30, 31], westwards across the Isthmus into the Darién [32, 33] after Spanish conquest.

### Survival of pre-Hispanic male lineages

Initially, it was thought that pre-Hispanic populations of the land-bridge would have high genetic diversity due to their intermediate position between complex cultures in the Andes and Mesoamerica. However, genetic studies using both classical [13, 34], mtDNA and nuclear, including Y-chromosome, markers [15, 35–42], demonstrate that these populations, mainly speaking Chibchan and Chocoan languages, are characterized by low genetic diversity and a high level of differentiation. In addition, no evidence of differential structuring between maternally and paternally inherited genes has been reported in isthmian Chibchan tribes [40, 41]. However, taking into account that the post-contact newcomers’ genetic contribution, especially for western Europeans, was mainly male-specific, a much lower male than female Native American component might be envisioned *a priori*. Moreover, in Panama, considering different degrees of geographical and genetic isolation during the past five centuries, this genetic component is expected to be found at a variable extent in different areas and communities.

In this study, in order to obtain a clearer picture of the complex genetic structure of the modern Panamanian population and to identify possible preferential mating patterns between local people and newcomers, we have investigated the Y-chromosome variation in 444 modern Panamanians, collected in eight of the nine provinces of the Republic and one indigenous territory (the comarca of Kuna Yala).

## Materials and Methods

### Ethics Statement

All experimental procedures and individual written informed consent, obtained from all donors, were reviewed and approved by the Comité Nacional de Bioética de la Investigación of Panama, by the Western Institutional Review Board, Olympia, Washington (USA) and by the Ethics Committee for Clinical Experimentation of the University of Pavia, Board minutes of October 5^th^, 2010.

### The sample

The total sample consists of 444 unrelated males who were part of the Perego et al. [3] dataset analysed for mtDNA variation. Out of these, 41 were included in the Battaglia et al. [43] dataset analysed for Y-chromosome haplogroup Q polymorphisms. Information relative to samples belonging to these datasets is reported in S1 Table. Samples were collected from healthy unrelated individuals in eight of the nine provinces and one indigenous comarca of Panama (Kuna Yala) (Table 1) in collaboration with the Gorgas Memorial Institute for Health Studies. Local helpers kindly assisted with the field sampling. Mouthwash rinsing was the primary method of biological specimen collection using a method (GenetiRinseTM) that comprises the use of 10cc of commercially available mint-flavored ScopeTM mouthwash in a 15cc volume leak-free NalgeneTM plastic container. Participants swished the 10cc of mouthwash for 45 seconds and then spat the mouthwash back into its original container. Participants were asked to abstain from eating or drinking for at least 30 minutes prior to the mouthwash rinse. Total DNA was extracted from mouthwash using standard commercial kits (Qiamp DNA Blood Maxi Kit, Qiagen) and stored at -20°C [3].

**Table 1:** Sample information.

Place of collection	N	PGF* birthplace	N
**Country**		**Country**	
Panama	444	Panama	408
		Foreign countries	24
		No information	12
**Province/Comarca**		**Province/Comarca**	
Bocas del Toro	21	Bocas del Toro	29
Chiriquí	80	Chiriquí	92
Coclé	25	Coclé	20
Colón	0	Colón	9
Darién	4	Darién	9
Herrera	31	Herrera	36
Kuna Yala	15	Kuna Yala	16
Los Santos	24	Los Santos	30
Panamá^#^	181	Panamá^#^	43
Veraguas	18	Veraguas	24
Not specified	45	Miscellaneous	100

* Paternal GrandFather

# Including Panama City, Panamá Oeste and Panamá Este districts

Sample pedigrees were analysed in detail in order to exclude subjects that might come from outside Panama and/or from the same family (distant relatives) on the paternal side. Due to the heterogeneity in the last known forebear ancestry on the paternal line—the terminal paternal ancestor (TPA)—of the 444 subjects in our sample, we decided to reallocate the samples by considering the paternal grandfather’s (PGF) birthplace. We found that for 12 subjects no information concerning the PGF birthplace was available while for 24 subjects the PGF was not from Panama (33% from Colombia, 25% from Central America, 25% from Europe, 17% from the USA). In agreement with data for the last known terminal maternal ancestors (TMAs) [3], none of the PGFs were reported as being born in Africa. After having removed these 36 samples, a total of 408 unrelated Panamanians were assigned to nine provinces and one comarca according to the PGF birthplace. One hundred samples lacking this information, but with PGFs born in Panama, were pooled into a group named “Miscellaneous” (S1 Table).

### STR genotyping

All samples were typed for 37 Y-STRs (33 single *locus*: DYS19, DYS388, DYS389I, DYS389B, DYS390, DYS391, DYS392, DYS393, DYS426, DYS437, DYS438, DYS439, DYS441, DYS442, DYS444, DYS445, DYS446, DYS447, DYS448, DYS449, DYS452, DYS454, DYS455, DYS456, DYS458, DYS460, DYS461, DYS462, DYS463, GGAAT1B07, YGATAA10, YGATAC4 and YGATAH4; four multi-*locus*: DYS385, YCAII, DYS459 and DYS464). They were analysed as described in Myres et al. [44] by means of custom designed amplification panels of multiplexed *loci* using fluorescent-labelled primers, capillary electrophoresis analysers with internal size standards, and quantitative fragment analysis software. Conversion of absolute fragment size to a number of allele repeats was achieved using the results obtained from sequencing both strands of CEPH control samples independently amplified with unlabelled primers.

### Haplogroup prediction analysis and its validation

According to the Y-chromosome phylogeny, haplogroups are defined by certain SNPs. On the other hand, different studies have demonstrated the existence of a clear correlation between haplogroups and STR haplotypes [24, 45–47]. Here the haplogroup classification was predicted on the basis of Y-STR *loci* information using two different predictor algorithms: the Haplogroup Predictor (http://www.hprg.com/hapest5/) with equal prior metapopulation and the YPredictor by Vadim Urasin (http://predictor.ydna.ru). The former software is based on genetic-distance [48] and Bayesian allele frequencies [45] calculated from collections of haplotypes extracted from published articles and public databases. The latter software is based on the phylogenetic trees of each haplogroup and uses the difference in marker values, marker mutation rates and age of parent nodes to calculate prediction probability. Both approaches assign a probability of haplogroup affiliation for a given Y-STR haplotype. The haplogroup assigned is the one that scored the highest probability value. It has been demonstrated that when at least 20 STRs are used, the prediction probability for the correct haplogroup can be higher than 99% in nearly all cases [49]. Thus, analyses were carried out with the 33 single STR *loci* haplotypes.

### Statistical analysis

Principal Coordinates (PCo) Analysis, on pairwise, individual-by-individual genetic distances, based on the 33 single STR *loci* haplotypes (S1 Table), was performed using Excel software implemented by GenAlEx 6.4 software [50].

Haplotype diversity and gene diversity over 15 (Yfiler haplotype, disregarding DYS385) and 33 *loci* were calculated using the software package Arlequin 3.5 [51].

Principal Components (PC) Analysis, based on haplogroup frequencies (S2 Table), was carried out using Excel through XLStat add-in. Populations from literature: Colombia (N = 308) [52], Costa Rica (Abrojo-Guaymí N = 19; Chorotega N = 23; Huetar-Zapatón N = 13) [23], El Salvador (Conchagua N = 23; Izalco N = 12; Panchimalco N = 11; San Alejo N = 12) [53], El Salvador Mestizo (N = 150) [54], Ecuador (Kichwa N = 91; Mestizo N = 99) [55], Guatemala (Maya N = 110; Mestizo N = 115) [56], Honduras (N = 128) [57] and Nicaragua Mestizo (N = 165) [46], Venezuela (N = 173) [58]. Haplogroup information was available only for Costa Rica and Nicaragua; for the other populations, the haplogroup prediction procedure previously described was performed.

Pairwise genetic distances (Rst values) and Analyses of MOlecular VAriance (AMOVA) were calculated for the Native American Q lineage using the eight Y-STR *loci* in common among Central American populations from literature (S3 Table). These, as well as haplotype diversity and unbiased gene diversity, were computed using the software package Arlequin 3.5 [51]. Rst distances were employed to generate a Multi-Dimensional Scaling (MDS) plot using Excel through XLStat add-in.

Median-Joining (MJ) network [59] was constructed using the Network 4.6.1.2 program (http://www.fluxus.engineering.com), after having processed data with the reduced-median method [60] and weighted the STR *loci* proportionally to the inverse of the repeat variance.

The ages of microsatellite variation were evaluated by using the evolutionary mutation rate of Zhivotovsky et al. [61] and the methodology already detailed in Battaglia et al. [43]. The rho statistic as implemented in Network 4.6.1.2 was also used (with the evolutionary mutation rate of Zhivotovsky et al.) [61] to estimate the coalescence of branches identified by Network analysis.

## Results and Discussion

### Haplogroup Analysis

Concordant predictions from the two algorithms were obtained for 393 out of 444 samples. Both predictors correctly assigned to haplogroup Q all the 41 M242-positive samples previously sub-classified by SNP analysis [43] (S1 Table). For 28 samples the two predictions, although not concordant, suggested two branches of the same haplogroup (E-M2/DE-YAP, E-M2/E-M123, J1-M267/J2-M172) or two haplogroups both mainly diffused in Asia (O-M175/H-M69). The remaining 23 subjects (5.2%), with discordant predictions from the two algorithms (S1 Table), were not classified into any haplogroup.

A total of 17 Y-chromosome haplogroups were identified in the 408 Panamanian samples with PGF in Panama (Table 2).

**Table 2:** Distribution of Y-chromosome haplogroups (%) in the Panamanian samples with PGF in Panama.

	Haplogroup																					
Province/Comarca (n. of subjects)	E-M2	E-M78	E-M81	E-M123	E-V13	G-P15	I-M253	I-P37.2	I-M436	J-M267	J-M172	O-M175	Q-M242	R-M198	R-M343 ^a^	R-M124	T-M184	DE-M1^b^	E-M96 ^c^	J-M304^d^	H-M69 / O-M175^e^	Non- predictable portion
**PANAMA (408)** ^**f**^	**4.7**	**0.2**	**0.2**	**3.9**	**2.2**	**2.5**	**1.5**	**2.9**	**1.5**	**2.0**	**7.4**	**1.2**	**21.8**	**1.2**	**31.6**	**0.5**	**2.9**	**1.0**	**4.4**	**0.5**	**0.5**	**5.4**
**Bocas del Toro (29)**				3.4							6.9	3.4	69.0		10.3	3.4			3.4			
**Chiriquí (92)**	4.3			5.4	1.1	2.2	1.1	2.2	2.2	1.1	6.5		18.5		39.1	1.1	1.1	1.1	6.5		2.2	4.3
**Veraguas (24)**				4.2			4.2				12.5		20.8	4.2	41.7				4.2	4.2		4.2
**Herrera (36)**	5.6			5.6	2.8	5.6		5.6		2.8	2.8		8.3	2.8	36.1		2.8		5.6			13.9
**Los Santos (30)**	3.3			3.3	3.3	6.7		3.3	3.3		16.7		6.7		23.3		10.0	6.7	3.3			10.0
**Coclé (20)**				5.0		10.0	5.0			5.0	5.0		30.0		35.0							5.0
**Colón (9)**			11.1					11.1					66.7		11.1							
**Panamá (43)**	9.3					2.3	2.3	7.0	7.0	2.3	7.0		14.0	2.3	32.6		7.0		2.3	2.3		2.3
**Kuna Yala (16)**					6.3								87.5				6.3					
**Darién (9)**	33.3										11.1		22.2					11.1	22.2			
**Miscellaneous (100)**	5.0	1.0		5.0	5.0	1.0	2.0	3.0		4.0	8.0	4.0	8.0	2.0	38.0		3.0		4.0			7.0

^a^ The great majority of these samples (122/142) were predicted as R-M269.

^b^ Samples predicted as DE-M1 or E-M40 by the Ypredictor and as E-M2 by the Hg predictor.

^c^ Samples predicted as E-M2 by the Hg predictor and as E-M123 by the Ypredictor.

^d^ Samples predicted as J1 by the Hg predictor and as J2 by the Ypredictor.

^e^ Samples predicted as H by the Ypredictor and as O by the Hg predictor.

^f^ Samples with PGF birthplace in Panama.

They were labelled according to the mutation-based nomenclature rules proposed by the Y Chromosome Consortium [62] and used by van Oven [63]: E-M2, E-M78, E-M81, E-M123, E-V13, G-P15, I-M253, I-P37.2, I-M436, J-M267, J-M172, O-M175, Q-M242, R-M198, R-M343, R-M124, T-M184. The haplogroup C-M130, which characterizes, mainly as C-P39, a minor portion of the Native American gene pool, was not identified by either predictors. However, due to the presence of 23 unclassified samples, its absence has to be taken with caution. The most frequent haplogroups are R-M343 (31.6%), Q (21.8%), E (15.6%) and J (9.9%). R-M343 shows its highest frequency in Chiriquí (39.1%); haplogroup Q represents the majority of the Kuna Yala (87.5%), Bocas del Toro (69.0%) and Colón (66.7%) samples; haplogroup E accounts for the majority (55.5%) of the small Darién sample, whereas haplogroup J shows a frequency higher than 15% only in Los Santos and Veraguas.

**Fig 1:**
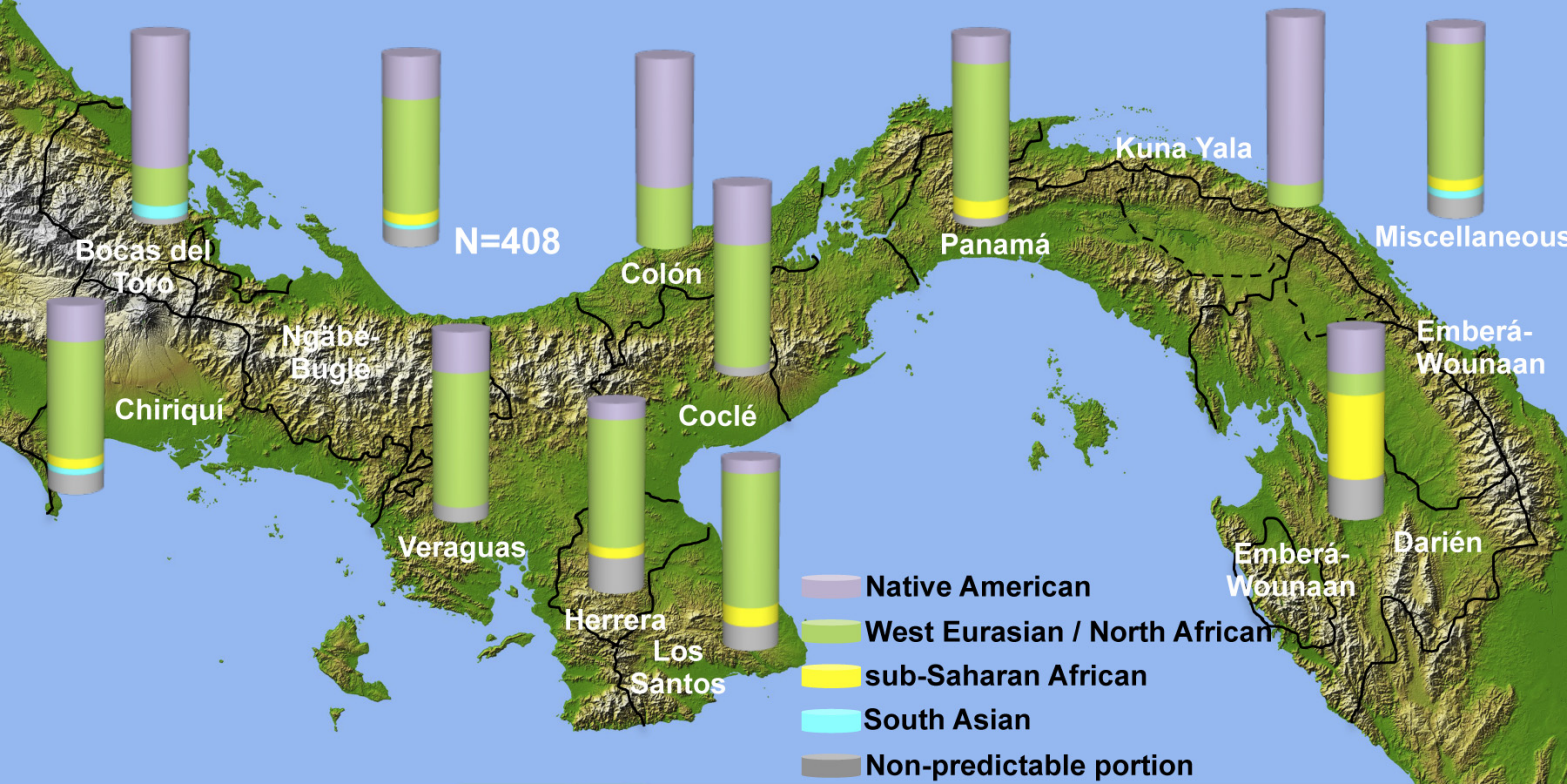
Spatial distributions of Y-chromosome components in Panama. Bars show Native American (violet), West Eurasian/North African (green), sub-Saharan African (yellow) and South Asian (light blue) components in each province or comarca. In grey the Y-chromosome portion with discordant haplogroup predictions. The physical map of Panama is from NASA Earth Observatory (http://earthobservatory.nasa.gov/).

When considering the geographical provenance of these haplogroups, four main genetic components (Native American, West Eurasian and North African, South Asian and sub-Saharan African) can be identified. The frequencies of these components *per* province are provided in Table 3 and illustrated in Fig 1.

**Table 3:** Y-chromosome components (%) in the Panamanian samples with PGF in Panama.

Province/Comarca	*Native American* ^*a*^	*West Eurasian and North African* ^*b*^	*sub-Saharan African* ^*c*^	*South Asian* ^*d*^	*Non- predictable portion* ^*e*^
(n. of subjects)					
**PANAMA (408)** ^**f**^	**21.8**	**60.3**	**5.9**	**2.2**	**9.8**
**Bocas del Toro (29)**	69.0	20.7		6.9	3.4
**Chiriquí (92)**	18.5	62.0	5.4	3.3	10.9
**Veraguas (24)**	20.8	70.8			8.3
**Herrera (36)**	8.3	66.7	5.6		19.4
**Los Santos (30)**	6.7	70.0	10.0		13.3
**Coclé (20)**	30.0	65.0			5.0
**Colón (9)**	66.7	33.3			
**Panamá (43)**	14.0	72.1	9.3		4.7
**Kuna Yala (16)**	87.5	12.5			
**Darién (9)**	22.2	11.1	44.4		22.2
**Miscellaneous (100)**	8.0	71.0	6.0	4.0	11.0

^a^ Hg Q-M242.

^b^ Hgs E-M123, E-M81, E-V13, G-M201, I-M253, I-P37.2, I-M436, J-M304, J-M267, J-M172, R-M198, R-M343, T-M184.

^c^ Hgs DE-M1, E-M2.

^d^ Hgs H-M69, O-M175, R-M124.

^e^ Samples with discordant haplogroup predictions (assigned to different haplogroups or to haplogroup sub-lineages that have discordant phylogeography).

^f^ Samples with PGF birthplace in Panama.

- The **Native American** component is represented only by the haplogroup Q-M242, presumably for the great majority by its sub-clade M3 [43], and accounts for 21.8% of the sample. Its frequency widely varies in the different provinces, ranging from 87.5% in Kuna Yala to 6.7% in Los Santos.
- The **West Eurasian and North African** component accounts for 60.3% of the Panamanian Y chromosomes. It is the most frequent component and includes the European haplogroups I-M253, I-P37.2, I-M436 [64, 65] and R-M343 [66], but also the Central-South European E-V13 [67–70], the Eurasiatic haplogroups R-M198 [71, 72], T-M184 [73] and G-M201 [74]. The Middle Eastern haplogroups J-M172 [75–77] and E-M123 [76, 78–80], along with the Middle Eastern/North African haplogroup J1-M267 [76, 81, 82] and the North African haplogroup E-M81 [76, 78] were also included in this component. With few exceptions (Bocas del Toro, Colón, Kuna Yala and Darién), the frequency of this component exceeds 50% in almost all provinces located on the Pacific side with the highest incidence in Veraguas, Herrera, Los Santos and Panamá.
- The **sub-Saharan African** component (5.9%) is characterized by the sub-Saharan African sub-clades of haplogroup E (in particular, E-M2) [78, 83, 84]. With the exception of the high frequency (44.4%) observed in Darién, its incidences range from 5% to 10% in Chiriquí, Herrera, Los Santos and Panamá provinces where during the Colonial period thousands of slaves were employed and where, before and after emancipation, they dispersed through the countryside. The sub-Saharan African component was not observed in Bocas del Toro, Veraguas, and Coclé, (Fig 1), three provinces which lay outside the trans-isthmian axis between Old Panama and the Caribbean ports of Colón that in different periods harboured important numbers of sub-Saharan African peoples. Although referred to a small size sample, the exceptionally high frequency of this component in Darién could represent the legacy of bands of escaped slaves, known as C*imarrones*, who were established in Darién during the latter part of the 16^th^ and early 17^th^ centuries and whose descendants still live along the rivers and in the coastal zones of this province.
- The **South Asian** component is the least frequent (2.2%) and it is represented by haplogroups H-M69, R-M124 [85] and O-M175 [86, 87]. This component was detected only in Bocas del Toro and Chiriquí as well as in the Miscellaneous group. Taking into account that the largest Chinese community of Central America is present in Panama, this component is likely to be present also in other provinces probably in the “non-predictable portion”. The background influence of Asiatic male lineages is the result of immigration of mostly southern Chinese and different peoples from the Indian sub-continent. These occurred after 1850 during the construction of the Panama Railroad and the French canal. Unlike the West Indians, however, the Chinese and people from British India set up businesses at this time, not only in the terminal cities, but also in Bocas del Toro, Darién, and the central provinces. Many Chinese and Indians became rich and influential citizens often inter-marrying with families of the Panamanian social elite [88]. Between 1913 and 1932, however, repressive legislation prohibited further immigration and curtailed the business activities of these groups [89, 90]. After the Second World War, this trend was reversed [90, 91].

### Haplotype Analysis

To explore the Y-chromosome haplotype variation in modern Panamanians, we carried out a PCoA based on the 33 STR *loci* haplotypes (S1 Table). S1 Fig illustrates the plot of the two principal coordinates (PCos) in relation to the predicted haplogroup affiliation. The two coordinates, which account for 63.7% of the total variance, identify three main Y-chromosome clusters: a Native American cluster, a West European specific cluster and a heterogeneous Eurasian and African cluster. On the first PCo, the Native American Y chromosomes occupy, along with few R-M198 and R-M124 Y chromosomes, an intermediate position between the West European cluster, which groups all the predicted R-M343 Y chromosomes, and the Eurasian and African cluster, which harbours all the remaining non Native Y chromosomes. On the other hand, the second PCo separates, with only a marginal overlap, Native American from all the other Y chromosomes and reveals a wide variation inside the Native American haplogroup Q, which suggests the existence of a not yet defined complex internal structuration.

The haplotype distribution *per* province (data not shown) underlines in almost all provinces a high heterogeneity, comparable to that of some other populations of the Isthmo-Colombian Area, which is confirmed by the population genetic parameters computed both for 33 and 15 (Yfiler disregarding DYS385) single-*locus* STRs (S4 Table). Not surprisingly, low values of gene diversity, similar to those observed in indigenous groups from Mexico [92], Argentina [93] and Bolivia [94], were registered in sampling units characterized by the highest frequency of haplogroup Q such as those of Kuna Yala and Bocas del Toro, two regions where indigenous populations offered active resistance to Spanish conquest and settlement, and continued pre-Spanish practices in mountainous and mostly forested terrains.

### Panama in the present-day Central American context

**Fig 2:**
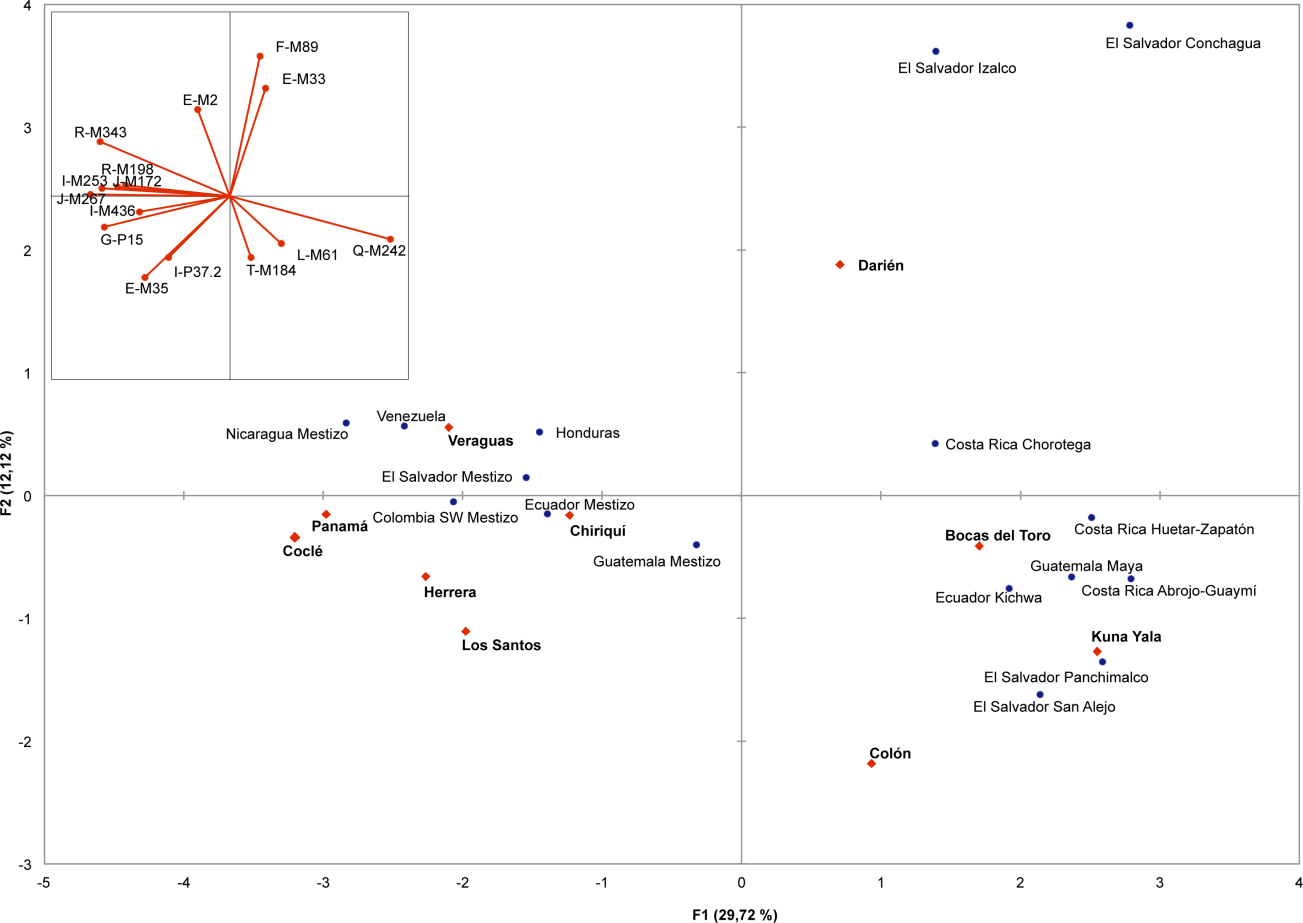
Principal Components (PC) plot based on the predicted Y-chromosome haplogroup frequencies. Numbers in parentheses indicate the proportion of the total genetic information retained by a given PC. The inset plot illustrates the contribution of each haplogroup. Red diamonds are for Panamanian groups; blue circles are for neighbouring countries.

Principal components analysis was performed comparing frequencies of the predicted haplogroups in the Panamanian population of the present study with those of neighbouring Central American populations from the literature (S2 Table). A plot of the first two components is illustrated in Fig 2.

Central American populations are distributed along the first PC mainly according to the proportion of Native American and West Eurasian components and along the second PC according to the different non Native American contributions. Thus, Kuna Yala, Bocas del Toro, Colón and Darién occupy, along the first PC, one extreme of the distribution together with Native populations from neighbouring countries, well separated from the Mestizo groups and the Panamanians from provinces facing the Pacific Coast. On the other hand, along the second PC, Panamá, Herrera, Chiriquí, Los Santos and, Coclé, displaying the highest frequencies of the sub-Saharan African component, are separated from Veraguas. This distribution pattern could reflect the violent behaviour of early Spanish incursions into the Pacific lowlands of Panama (1515–1530) including the eastern side of the Azuero Peninsula, where the regional population was large and concentrated in vulnerable nucleated villages along major rivers. This resulted in rapid depopulation enhanced by enslavement and forced relocation elsewhere, disproportionately high male mortality and the movement of survivors to still autonomous areas [95–97].

### Analysis of the variation inside the Native American haplogroup Q

**Fig 3:**
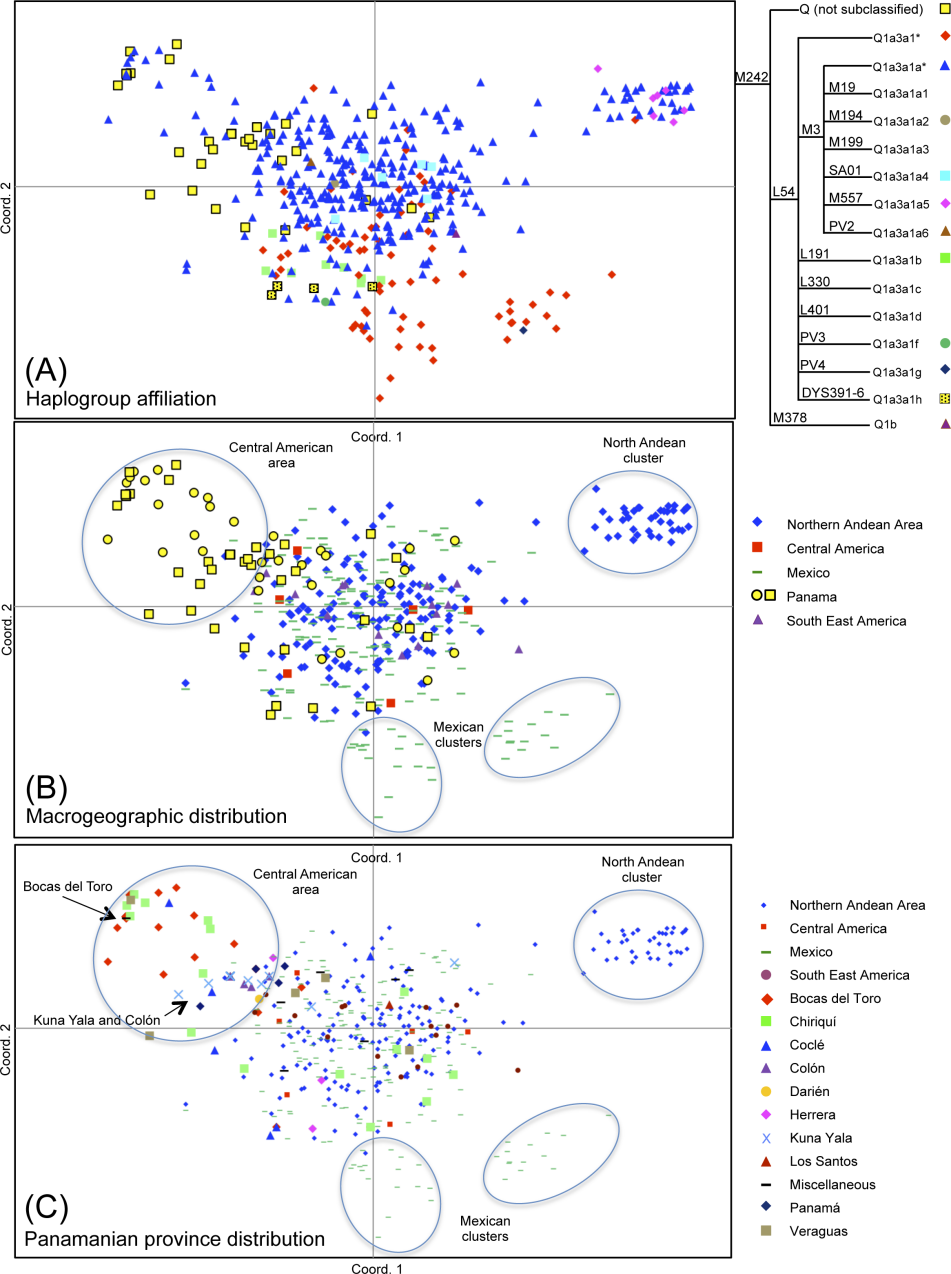
Principal Coordinates (PCo) plots. Analysis was performed on the 33 STR *loci* haplotypes (S1 Table) screened here together with those previously typed by Battaglia et al. [43]. On the whole, 46.13% of the total variance is represented: 25.62% by the first PCo and 20.51% by the second PCo. Plots are in relation to haplogroup affiliation (A), macrogeographic distribution (B), and Panamanian province distribution in the Central-South American context (C). In panel A, yellow squares are the Panamanian Y chromosomes predicted as belonging to haplogroup Q and not further sub-classified; dotted yellow squares indicate six samples (two of these are completely overlapping) characterized by the DYS391-6 allele. The tree on the right illustrates the phylogenetic relationships among haplogroups. Nomenclature is in agreement with Battaglia et al. [43]. In panel B, Panamanian samples are represented by yellow symbols: squares are for predicted haplotypes and circles for haplotypes that were instead classified by SNP analysis [43]. South East America includes Argentina, Brazil, Paraguay and Uruguay; Central America includes Costa Rica, El Salvador, Guatemala and Nicaragua [43].

To evaluate the variation of haplogroup Q in Panama in comparison with neighbouring regions, a PCoA of the 33 STR *loci* haplotypes observed in our sample (S1 Table) was performed together with those previously examined by Battaglia et al. [43] and representative of Central America (mainly from Mexico) and South America (mainly from the northern Andean region) (S5 Table). The plots of the two principal coordinates (PCos) in relation to haplogroup affiliation (panel A) and geographic distribution (panels B and C) are shown in Fig 3.

The first principal coordinate distinguishes a Central American area, consisting exclusively of Panamanian samples, and a compact northern Andean cluster, from the bulk of samples located in the centre of the plot (Fig 3). The second coordinate distinguishes two Mexican Y-chromosome groups, separating the haplotypes related to the M3 background (Fig 3A, upper part of the plot) from haplotypes associated with the L54(xM3) context. The separation between the most frequent M3 Y chromosomes (blue triangles in Fig 3A) and the less represented L54* (red diamonds in Fig 3A) allows to hypothesise that the great majority of Panamanian Y chromosomes of the present study, predicted as belonging to haplogroup Q (yellow squares in Fig 3B), belong to the M3 lineage, in agreement with the results obtained by Battaglia et al. [43]. By comparing the Panamanian haplotype Q distribution with that of Central and South Americans (Fig 3B), no separation emerges in the central area of the plot among Panamanian, Mexican and Andean Y chromosomes. However, specific Panamanian, Andean and Mexican clusters at the fringes of the plot are evident. While the lack of differentiation in the central area of the plot can be ascribed to the common genetic background derived from an initial strong founder effect, such clusters indicate local differentiations that persist despite documented ancient and recent contacts among these areas. Since the dawn of agriculture, domesticated plants moved into Panama from Mexico and the northern Andes, but probably indirectly through reticulate exchange networks among small farming communities. Subsequently, artisans in the employ of Panamanian chiefs may have arrived from the gold-working areas of Colombia including the Andes. In addition, archaeological and ethnohistoric evidence testifies frequent trading contacts between Panama and Mexico (including the Maya world) during the last 1,500 years of the pre-Colombian era, mostly along the Caribbean coast.

**Fig 4:**
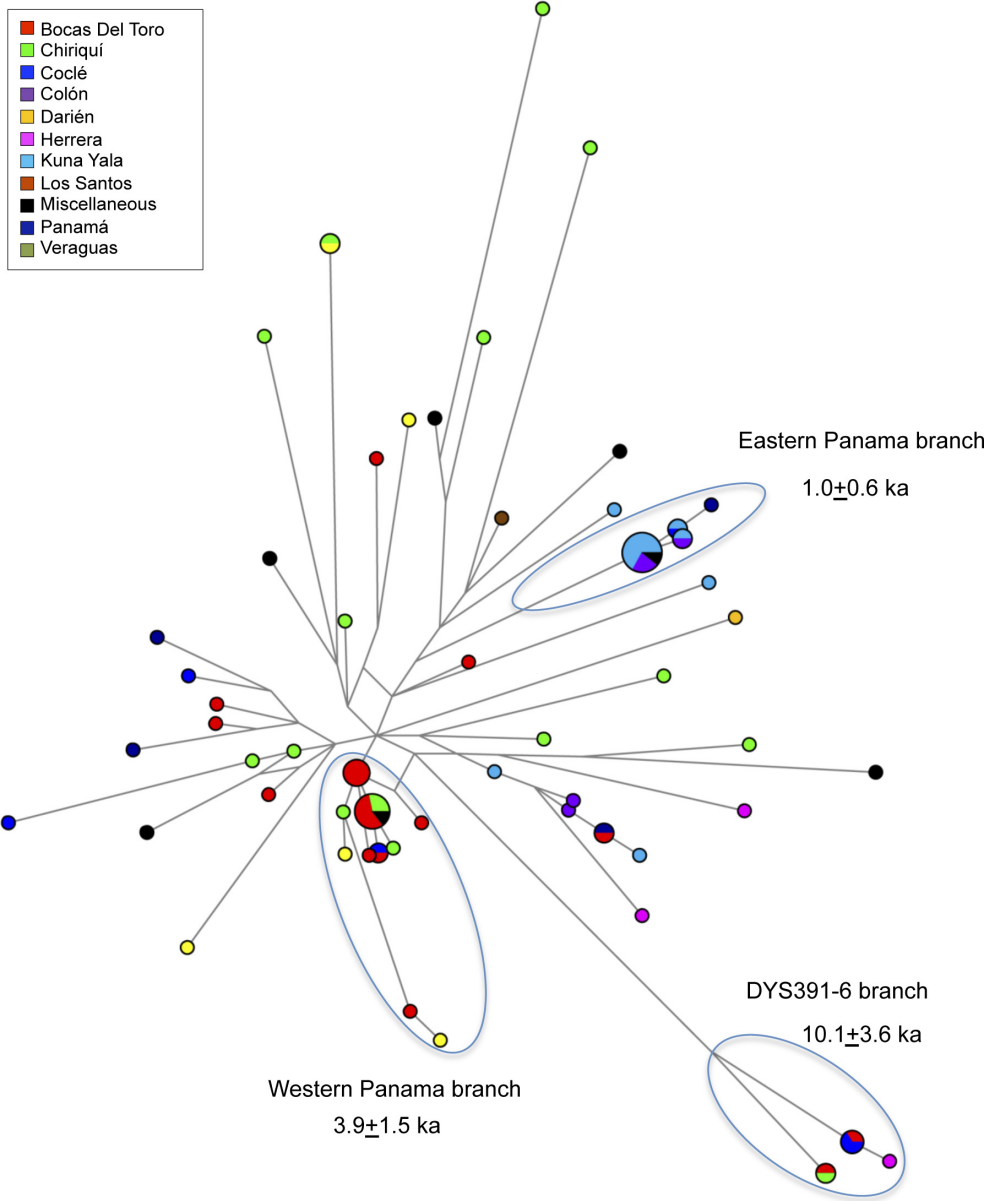
Network of Panamanian Q STR haplotypes. The analysis was performed on 15 *loci* (S1 Table). Samples were subdivided according to their PGF birthplaces, marked by different colours. The size of each circle is proportional to the haplotype frequency; the smallest circle is equal to one subject. The coalescence time estimates of the three branches were obtained by using the rho statistic and the 15 STRs *loci* considered in this analysis.

When the province of each Panamanian subject is considered (Fig 3C), samples from Chiriquí, Veraguas and Coclé are present both in the M3 and M242(xM3) areas of the plot whereas Kuna Yala, Colón and Bocas del Toro display a substantial presence of Q-M3 Y chromosomes. These M3 chromosomes, although part of the same Central American area (Fig 3C), are clearly differentiated by Network analysis (Fig 4) into two well separate branches: one, the Eastern Panama branch, made up virtually only by Kuna Yala and Colón subjects and the other, the Western Panama branch, including mainly Bocas del Toro subjects.

A further potential interesting branch is that defined by the peculiar 6 repeat allele at *locus* DYS391. This allele, which falls into the L54(xM3) context [43] (S1 Table and Fig 3A), was described for the first time in five subjects from Panama [41]. Subsequently, it was observed in eight additional samples, one from Panama, five from Colombia [43, 52] and two from Nicaragua [24]. Our detection of four additional Y chromosomes carrying the DYS391-6 repeat allele led us to better investigate its distribution and associated microsatellite variation. Although very rare (only 210 observations out of 136,184 subjects of the YHRD database-release 48; http://www.yhrd.org [98], this allele has been observed only in populations from Central and South America (24 Y chromosomes, 62.5% from Colombia), and East Asia, mainly belonging to the Han ethnic group, and South East Asia. The strong differences at various *loci* for the STR haplotypes associated with this allele in Asians and Native Americans and the lack of Q-L54* in Asia so far, lend support to the scenario that these Asian and Amerindian Y chromosomes represent two independent lineages. Furthermore, although only few samples were investigated for haplogroup affiliation and classified as Q-P36(xM3) (five subjects in Panama) [41], Q-M346 (two subjects in Nicaragua) [24] and Q-L54 (one subject in Panama and one in Colombia) [43], it is reasonable to hypothesise that the appearance of this allele in the Americas occurred in the Q-L54 main founding lineage. In addition, taking into account the internal variation of the Q-DYS391-6 lineage (Var = 0.399; Age = 14.5±5.2 ka), it is likely that it originated during the early stages of the continent peopling, thus indicating that the L54 haplogroup participated in the early and rapid colonization process of Central and South America crossing the Panama land bridge, and underwent processes of local differentiation in both Mexico (L191 and PV3) [43] and the Isthmo-Colombian Area (DYS391-6). Finally, by considering these new data, it emerges that within the Isthmo-Colombian Area the Q-DYS391-6 sub-lineage is less variable in Panama (Var = 0.191; Age = 7.5±3.1 ka) than in Colombia (Var = 0.316; Age = 14.9±4.5 ka) (Table 4). On the other hand, also the network of the associated haplotypes (S2 Fig), which branches out from a haplotype observed in Colombia, would, at first glance, suggest that the presence of Q-DYS391-6 lineage in Panama is ascribable to back migrations from the northernmost South America [99]. However, when the two Nicaraguan samples Q-DYS391-6 [24] are considered, this lineage reaches in Central America a value (Var = 0.331; Age = 14.2±7.2 ka) comparable to that estimated in Colombia. Thus, in light of these considerations, the Q-DYS391-6 lineage could be the result of an ancient differentiation of the Q-L54 founding lineage in the Isthmo-Colombian Area and the present variation in the different regions of this area could reflect more recent local demographic events: genetic drift, isolation, as well as a reorganization of Isthmo-Colombian Area populations largely speaking Chibchan languages. Indeed, as illustrated by the network (S2 Fig), the lower variation registered in Panama is due to a local diffusion of one haplotype.

**Table 4:** Estimated coalescent ages^a^ for the Q-DYS391-6 lineage in Central and South America.

Country (n. of subjects)	Age ± SE	Variance
DYS391-6 (30)^b^	14.5±5.2	0.399
Panama (12)	7.5±3.1^c^	0.191
Colombia (15)	14.9±4.5	0.316
Central America (14)	14.2±7.2	0.331
South America (16)	12.1±4.2	0.305

^a^ Only the six Y-STR *loci* in common with comparison population samples from literature are considered (S6 Table).

^b^ Samples from Battaglia et al. [43]; Núñez et al. [24]; Julieta Ávila et al. [52]; Ascunce et al. [41]; YRHD database [98] (S6 Table).

^c^ The estimate obtained for the six Y chromosomes of this study by using the rho statistic and 15 STRs *loci* provides a value within this range (see Fig 4).

On the whole, three well-structured branches were identified by Network analysis (Fig 4): the Western Panama branch and the DYS391-6 branch, both dating > 3 ka ago, could represent the legacy of descendants of populations that have lived continuously in some areas of Panama for a very long time [6, 13] whereas the Eastern Panama branch, dating to post-Columbian times, could represent the result of an expansion after the bottleneck determined by the European arrival.

**Fig 5:**
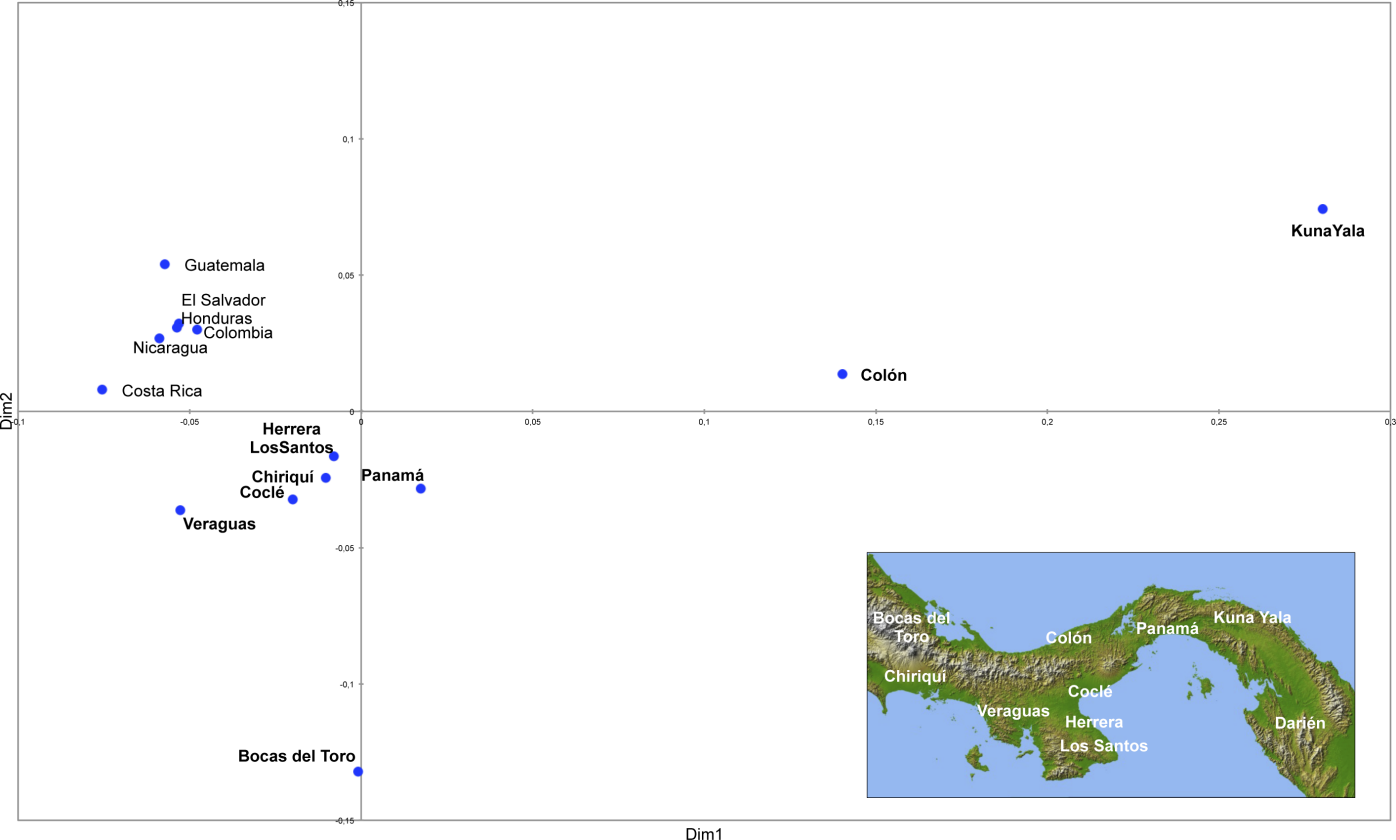
MultiDimensional Scaling (MDS) plot. Analysis was based on the pairwise R_ST_ genetic distance matrix calculated on eight STR *loci* haplotypes of haplogroup Q samples from Panama and neighbouring countries (S3 Table). Panamanian provinces are indicated in bold. The neighbouring provinces of Herrera and Los Santos were pooled to reach the minimum number of four samples per considered area. The province of Darién was not considered, being represented by only one sample. Stress value: 0.251. In the inset, the physical map of Panama (from NASA Earth Observatory: http://earthobservatory.nasa.gov/).

Bearing in mind the variation observed inside haplogroup Q, Panamanian Native American demography was investigated through MDS analysis over Rst distances (Fig 5) on the most represented groups of Panama and on the neighbouring countries of the Isthmo-Colombian Area (S1 and S3 Tables).

The provinces of Kuna Yala, Colón and Bocas del Toro, displaying virtually only haplotypes of the Central American M3 cluster (Fig 3), are well separated from each other and from all the Pacific provinces. This separation, which is confirmed by AMOVA analysis identifying 21.7% of variation (p value<0.01) among these groups (S7 Table), could be ascribed to a different proportion of the two main Native American Q lineages, Q-L54 and Q-M3. The diffusion of the less frequent Q-L54 seems to be limited to the Pacific provinces, despite the general lower presence in this area of the Native American component (Fig 1). On the other hand, the more frequent Q-M3 is widespread and is the most represented toward the Caribbean slope.

### Combined analysis of Y-chromosome and mtDNA haplogroups

**Fig 6:**
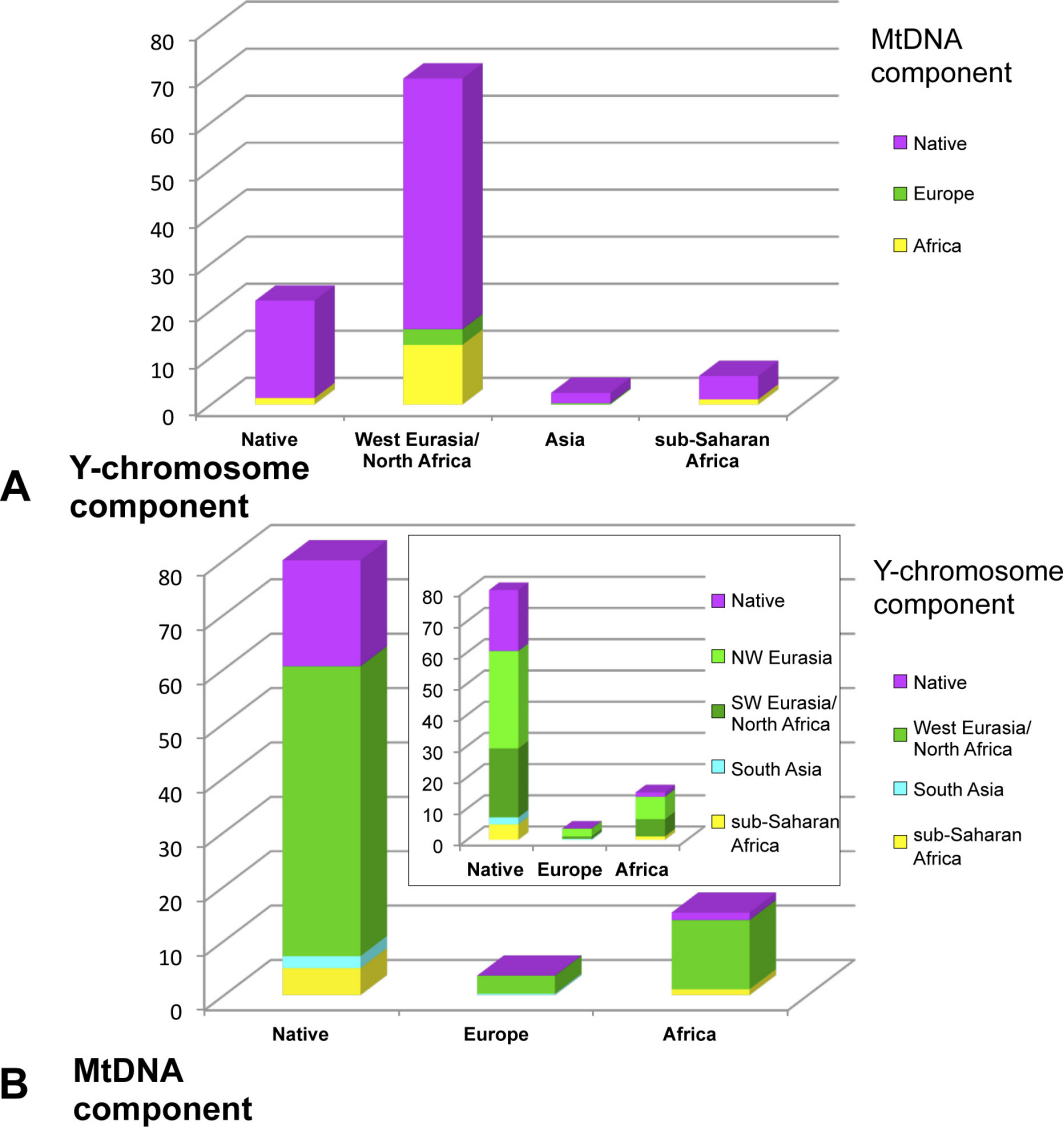
Combined analysis of Y-chromosome and mtDNA components. Analysis was performed on the male individuals classified in the present paper for Y-chromosome haplogroups and by Perego et al.[3] for mtDNA haplogroups (S1 Table). (A): along the X-axis subjects are classified on the basis of the Y-chromosome component they belong to; on the Y-axis the coloured portions of the histogram indicate the percentage of each mtDNA component (*e*.*g*. almost the totality of males carrying a Native Y-chromosome haplogroup have also a Native mtDNA). (B): on the X-axis subjects are classified according to the mtDNA component they belong to; along the Y-axis coloured portions indicate the percentage of each Y-chromosome component. In the inset plot the Eurasian component has been split into North West Eurasian and South West Eurasian / North African components.

The Y-chromosome analysis of Panamanians was performed in the males of the larger sample analysed for the mtDNA variation by Perego et al. [3]. Thus, by using both mtDNA and Y-chromosome information (S1 Table), it was possible to investigate the combined maternal and paternal ancestry for our samples (Fig 6). The near totality of male subjects have a mtDNA ancestry (female ancestor) in America, as already observed in Perego et al. [3]. In particular, virtually all males carrying Native, Asian and sub-Saharan African Y-chromosome haplotypes (horizontal axis in Fig 6A) show Native mtDNA haplotypes (violet portions of histograms). As for those subjects belonging to West Eurasian and North African Y-chromosome haplogroups, about 13% of them show an African mtDNA ancestry and very few a European one. By considering the reciprocal analysis of mtDNA (horizontal axis) *versus* Y-chromosome (vertical axis) haplogroup assignment, the result looks very different with a large prevalence of Y-chromosome haplogroups (male ancestry) from Eurasia (Fig 6B, green portions of histograms).

The comparative analysis of the Panamanian mtDNA and Y-chromosome lineages clearly shows a strong unidirectional sex bias in European-Native American admixture. This phenomenon, which has already been described in Central American areas [24], is most likely due to a first asymmetric mating between male newcomers and Native American women followed by preferential mating between Mestizo women and European males. Consequently, in the face of a substantial decrease of Native American Y-chromosome (paternal) component, a major Native American mtDNA (maternal) component has been retained. In addition, high male mortality in unequal conflicts, and forced transportation of surviving males to Panamanian mines and to other areas subjugated in the early Spanish Empire, including Peru, consolidated the female bias of post-contact Native American survival.

## Conclusions

In this study we provide an overview of the Panamanian Y-chromosome gene pool. The Y-chromosome perspective differs from that of mtDNA, which revealed a predominant (83%) Native American component [3]. Indeed, the Native American Y-chromosome component exceeds 50% only in three populations facing the Caribbean Sea: the comarca of Kuna Yala and Bocas del Toro province where Chibchan languages are the majority, and the province of Colón where many Kuna and people of mixed indigenous-African and-European descent live. In the other provinces the West Eurasian component is very high (> 65%; with the only notable exception of Darién, see below) and is mostly represented by European haplogroups, as a signature of the strong male genetic impact of European invaders and/or more recent European demographic influences. The sub-Saharan component (5.9%) identified in the Pacific provinces of Panamá, Chiriquí, Herrera, Los Santos and particularly in Darién represents the legacy of the African slaves who first entered Panama at the beginning of the 16^th^ century and were used in gold mines, pearl fisheries, ranching and agriculture, and military activities.

The preponderance of the main founding lineage Q-M3 and its haplotype differentiation (Fig 3), which only partially overlaps that of South America [43], shows that Panama after being fairly rapidly and continuously inhabited since the first arrival of Paleo-Indians underwent a local differentiation. This scenario is supported also by the presence in Panama of the Q-L54 haplogroup and its Q-DYS391-6 sub-lineage, which likely differentiated in the Isthmo-Colombian Area before 10 ka ago.

## Supporting Information

S1 Fig Principal Coordinates (PCo) plots. Analysis was performed on Panamanian Y-STR haplotypes (S1 Table) based on pairwise, individual-by-individual genetic distances related to haplogroup affiliation. On the whole, 63.75% of the total variance is represented: 41.16% by the first PC and 22.59% by the second PC.
(TIF)

S2 Fig Network of the STR haplotypes associated with the Q-DYS391-6 lineage. The analysis was performed on six STR *loci* (S6 Table). The size of each circle is proportional to the haplotype frequency; the smallest circle is equal to one subject. Different countries are marked by different colours.
(TIF)

S1 File History and linguistic details. (DOCX)

S1 Table Haplogroup/sub-haplogroup classification for the 444 Y male samples collected in Panama. (XLSX)

S2 Table Absolute frequencies of Y-chromosome haplogroups and sub-haplogroups in the 26 populations included in the PCA. (XLSX)

S3 Table List of the Y-STR haplotypes in common among the Central American populations considered in the AMOVA and MDS analyses. (XLSX)

S4 Table Population genetics parameters for the Panamanian samples with 33 and 15 Y-STR *loci* haplotypes. (XLSX)

S5 Table List of the Y-STR haplotypes previously reported by Battaglia et al. (2013) and considered in the PCoA plot of Fig 3. (XLSX)

S6 Table Y-chromosome haplotypes associated with the DYS391-6 allele. (XLSX)

S7 Table AMOVA analysis carried out on Q haplotypes. (XLSX)
