# Supplementary figures and images for: Exploring the Y Chromosomal Ancestry of Modern Panamanians
Source: PLoS One. 2015 Dec 4;10(12):e0144223. doi: 10.1371/journal.pone.0144223 (PMC4670172; doi:10.1371/journal.pone.0144223)

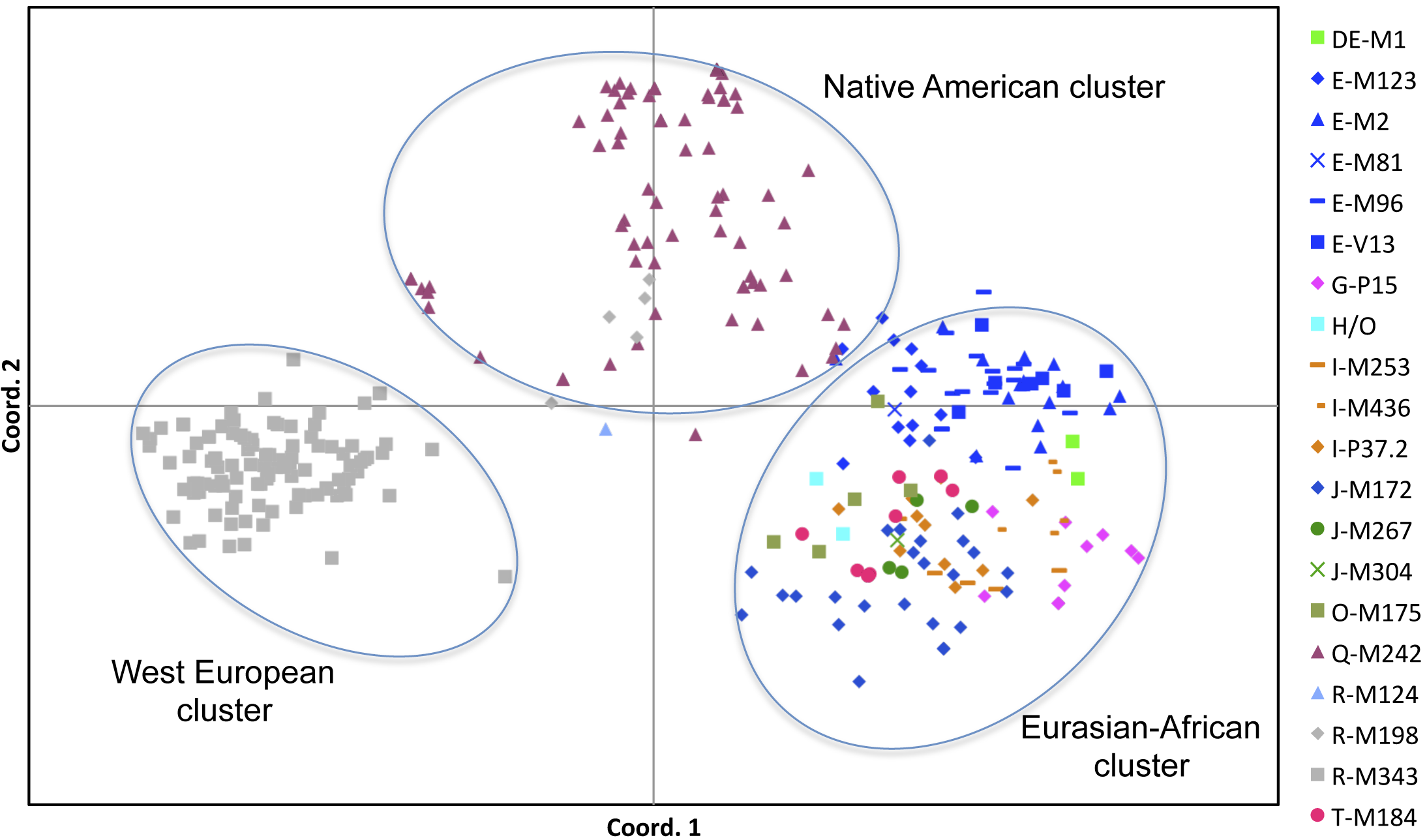

Supplement: S1 Fig — Analysis was performed on Panamanian Y-STR haplotypes (S1 Table) based on pairwise, individual-by-individual genetic distances related to haplogroup affiliation. On the whole, 63.75% of the total variance is represented: 41.16% by the first PC and 22.59% by the second PC. (TIF) [file pone.0144223.s001.tif]

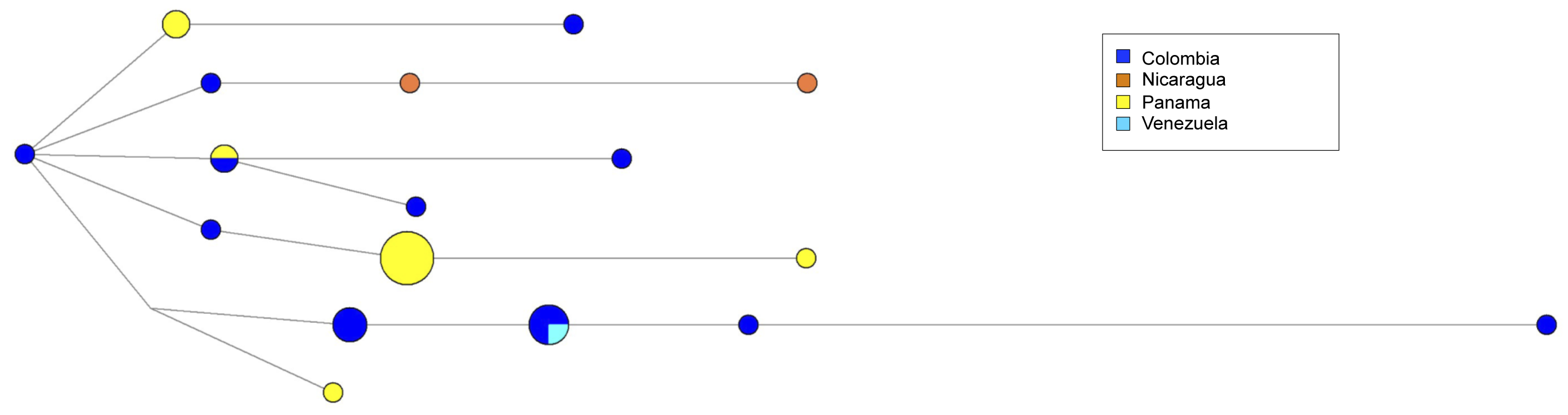

Supplement: S2 Fig — The analysis was performed on six STR loci (S6 Table). The size of each circle is proportional to the haplotype frequency; the smallest circle is equal to one subject. Different countries are marked by different colours. (TIF) [file pone.0144223.s002.tif]
