# Supplementary material for: Exploring the Y Chromosomal Ancestry of Modern Panamanians
Source: PLoS One. 2015 Dec 4;10(12):e0144223. doi: 10.1371/journal.pone.0144223 (PMC4670172; doi:10.1371/journal.pone.0144223)
Supplement: S1 File — (DOCX) [file pone.0144223.s003.docx]

## Antiquity and coherence of the Chibchan heritage

The majority of surviving and recently extinct languages spoken in the Isthmo-Colombian Area are Chibchan. It is hypothesized, on the basis of the theory of linguistic affinity decay, [1-3] that the Chibchan proto-language coalesced about 10,000 14C years ago in a “core area” on the lower Central American isthmus (southern Costa Rica and western and central Panama), and subsequently radiated into three branches: western (“Votic”), central (“Isthmic”) and eastern (“Magdalenian”). Each branch then underwent further fissioning. It is argued whether the Magdalenian branch began to fission before or after the initial Chibchan movement into northern Colombia.

Genetic data including dermatoglyphics strongly support the Constenla linguistic hypothesis and an earlier one by Hershkowitz [4-9]. Archaeologists, documentary historians, ethnohistorians, paleoecologists, and population geneticists make a strong consensual case for considering the Isthmo-Colombian Area to be a coherent phylogenetic and cultural unit throughout the pre-Columbian era, differentiated from the rest of South America [10]. Nevertheless it is important to beware of the dangers inherent in comparing data with dissimilar methodologies that use different methods of estimating the passage of time. The long-term history of native peoples in the Isthmo-Colombian Area, however, shows important differences from that of the vast continent of South America, where, east of the Andes, few mountain ranges constitute real barriers to movement. Besides, this region underwent a very different post-contact history characterized by extensive displacements [11]. Thus the utility of comparing the human genetic and linguistic histories of “Central America” as an entity distinct from “South America”, following modern geopolitical divisions, is questioned.

## Diversification and exchange networks

Socio-political fragmentation across the Chibchan Core area (Costa Rica excluding Guanacaste and western and central Panama) is epitomized in the pre-Columbian era by semi-monumental ceremonial-cum-social centres. Many of these exhibit idiosyncratic features suggestive of group identity (e.g., stylistically distinctive statuary and site-specific architecture and layouts) [12-16]. Chiefdom-formation encouraged and diversified exchange. Most exchange was carried out within local and regional networks especially among contrasting biomes on both watersheds and in different vegetation formations [12, 17, 18]. Longer-distance exchange was driven by the elites’ demands for exotic raw materials and objects, particularly those made of cut and polished jade and jadeite and silica-rich stones [12]. This kind of interaction benefitted from the terrestrial connections of the lower isthmian land-bridge and also from coastal connections by canoe particularly along the Caribbean marine littoral that lacks extensive mangrove-estuary systems. Maritime connections between the Pacific mainland and the Pearl Islands, 50 km away in Panama Bay, are documented for the period 6,200-5,600 years BP [19]. Colonies of Mesoamerican traders seeking mostly gold alloy artefacts and cacao, are documented by Spanish chronicles just before and just after contact, and probably existed long before this [18, 20, 21]. On the contrary, pre-contact exchange interactions between the isthmus and the northern Andes are not well documented by archaeology. Emeralds were one product that was most likely obtained in the northern Andes[18]. Thorny oyster (*Spondylus*) shell-work and cast gold-alloy figurines are more ancient in Colombia and Ecuador than in Panama and Costa Rica although isthmian artisans working in both media quickly developed technological and iconographic idiosyncrasies after these technologies entered Panama about 2,000 years BP [22, 23]. High carat gold ores, copper used to fabricate alloys, and *Spondylus* shells are widely distributed in Panama. Even though the introduction of some important technologies followed a clear south-north or north-south trajectory (in the cases of pottery, metal-working and polished stone jewellery), no evidence indicates that these were accompanied by sizable population movements. Once new technologies appeared on the land-bridge, they adapted quickly to locally available raw materials and tastes [12, 17, 18, 22]. Some, like the Monagrillo pottery ware, are hypothesised to have been local (independent) inventions even though the technology itself appeared earlier in South America [24].

## Native American resistance and survival

The troops responsible for the destructive impacts of Spanish conquest on the populous savannah-lands of the isthmian Pacific watershed were sent out from Santa María la Antigua (Gulf of Urabá), Acla in Kuna Yala, Old Panama, and from Natá in Coclé (the first Spanish towns founded in the west) [25, 26]. By 1550 some pre-Hispanic social units across this area had effectively disappeared [25-28].

Native American resistance thereafter did not follow a standardized pattern. In Nicoya (north-west Costa Rica) and south-western Nicaragua, polities that had entered these areas from Mesoamerica after 1,300 years BP retained some of their pre-Hispanic social structures until well into the Colonial period [29]. Other areas of the land-bridge remained difficult for the Spanish to penetrate even though some presented desirable economic resources, such as gold [30]. In Caribbean Costa Rica and western Panama, which remained extensively forested, native resistance was more effective inducing a higher degree of survival of pre-Hispanic customs, such as head-taking, and making and using cast copper-gold ornaments [31, 32].

Important post-contact movements of specific Chibchan cultural groups have been documented in Panama for (1) the Naso Djërdi, from the western Caribbean coast to the Térraba valley on the Pacific watershed, (2) the Kuna, from the Tuyra and Chucunaque valleys towards the lower Atrato drainage and the San Blas coast (Kuna Yala) and islands. The Chocoan-speaking Emberá and Wounaan expanded into Darién and, more recently, eastern Colón province and eastern Panama mostly from the Atrato, Baudo and San Juan drainages [33-35]. They entered into contact with the Kuna. Much debate has centred around a polity identified by the Spanish as the “Cueva” whose settlements extended from the eastern slopes of the El Valle volcano (Coclé and Colón) to the western shore of the Gulf of Urabá and the Gulf of San Miguel on the Pacific. The term “Cueva”, however, does not refer to an ethnic group, but rather to a language whose features and distribution suggest it was either a trade language similar to the Chibchan Huetar language and to many others in polyglot regions of the world, or a group of vernaculars whose linguistic history and coherence are impossible to ascertain from the limited documentary evidence at hand. The “Cueva” vocabulary of about 55 words, which were recorded in Spanish chronicles written in the 16^th^ century, includes some words that are clearly cognate with every-day Chocoan usage today. In addition, contact-period toponymy in eastern Panamá and Darién provinces makes ample use of the terminal phoneme - ra (a denominator of “person” in Chocoan languages). Since Chibchan and Chocoan peoples are differentiated at the mtDNA, nuclear DNA and Y-chromosome levels the possible presence of ancestral Chocoans in eastern Panamá province and Darién since before the Spanish contact should be contemplated [33, 36].

Post-contact movements of surviving Native American groups have been well documented in western Panama (Bocas del Toro) and eastern Costa Rica. Most were short-distance (<100 km). Some were trans-cordilleran as in the case of the Naso-Djërdi (or Teribe/Térraba). Many relocations were promoted by religious organizations [26]. Others were voluntary being driven by the desire to move away from Spanish influence, to establish trade relations with Europeans, and to escape the marauding Mískito, a mestizoised ehtnic group on the Caribbean that spoke a non-Chibchan (Misumalpan) language [37]. Active military resistance was offered until the 17^th^ century by the Coclé (an eastern branch of the Ngäbe) [37] in the central Caribbean, and by the now extinct Doraces who lived in Bocas del Toro.

Lastly, population genetic studies pass over two lines of evidence. The first is the dispersal across central and western Panama of male and female Native American slaves from outside Panama, many of these from Cubagua (Venezuela) and Nicaragua, with smaller numbers from Peru. The second is the keeping of male and female indigenous slaves by surviving native Chibchan groups in Caribbean Panama and Costa Rica up to the 19^th^ century. This resulted in short-distance movement of individuals among different small ethnic groups [38].

## The isthmian heritage of the Kuna, Ngäbe and Bugle

A 62.5% presence of the mitochondrial A2af sub-lineage in the modern Kuna Yala population infers that the Kuna’s remote heritage is isthmian notwithstanding their post-conquest settlement focus in the border regions of Panama and Colombia [34, 39, 40]**.** Constenla-Umaña’s historical linguistic hypothesis is supportive [2]. He places the Kuna language in an eastern isthmian clade along with the languages of the Bugle and Ngäbe. He inferred that this clade coalesced 4,800 years BP, and also that the Kuna language separated from it soon after [2, 3].

Ethnopharmacogenetic studies since the 1980s [41], in addition to recent re-analyses of the genetic and cultural history of a tri-hybrid post-conquest Caribbean population in central Caribbean Panama (the so-called “Cholos de Coclé”), have provided evidence for genetic connections between the Kuna and the Ngäbe. Two gene variants are shared by the hybridised Coclé and their recent ancestors, the Ngäbe: LDHb-GUA, a private polymorphism, and TFD-GUA. The hybrid “Cholos de Coclé” also possess the PEPA-KUNA gene variant, which presumably came into their population through contact with the Kuna. None of these markers has been detected among the Bugle [37]. The Bugle were often called “Guaymí Sabanero” in documents of the 18th. and 19th, centuries, i.e., “Guaymí” who lived in the savannas of the Pacific watershed, as distinct from the Ngäbe, whose historic settlements stretched along the Caribbean coast from Almirante Bay to the Cricamola River [26, 42, 43]. After conquest, the “sabanero” population split into three sub-groups, with two enclaves remaining on the Pacific side and a larger enclave (frequently named “Bokotá”), which moved across the central mountain chain into the Calovébora River valley and affluents [42] in the central Caribbean watershed where several communities exist today [44].

The Kuna are identified in Spanish documents by many names. Their attacks against Spanish forts and settlements started at the beginning of the 17^th^ century and reached as far west as Chepo, an acculturated native American settlement now in eastern Panamá province. In 1617 a “Cuna-Cuna” chief was received by the governor of Cartagena. A Flemish Franciscan friar, Fray Adrián de Uffeldre, resided with the Kuna between 1630 and 1640, observed the elevated percentage of albinism that characterizes this cultural group, and recorded ceremonies of passage and a vocabulary, which are recognisably Kuna. There followed a long period of oscillating relations with the ecclesiastical and Crown authorities: successful and unsuccessful indoctrination attempts, armed risings, treaties made and broken, and the destruction of towns with much loss of life. The outcome was that the Kuna achieved a considerable degree of independence, which they have maintained to this day. They also entered into relations with English, Scottish and French pirates, traders and settlers [40, 45].

The Kuna themselves consider their original homeland to comprise the lower Atrato basin, the southern shore of the Gulf of Urabá, and the headwaters of the large Darién rivers (Chucunaque and Tuyra) [40]. This idea is supported by Kuna oral history and myths, and by fluvial toponymy current in early Colonial times: the phonemal suffix *–ti* in Kuna signifies ‘river’ whereas in Chocoan languages it is *–do*. After the movement of the Kuna population to the Kuna Yala (“San Blas”) coast in the 19^th^ century, some villages remained in the afore-mentioned areas where they entered into contact with mostly French settlers. This geographic separation led to dialectal differentiation in the Kuna language although physical contact among these Kuna communities has continued. Inland Kuna savants are considered to be exceptionally well versed in Kuna history and lore. This fact influenced Mary Helms’ hypothesis of long-distance journeys made by Kuna coastal leaders in order to become imbued with esoteric knowledge [46]. In the context of isthmian geography, “long-distance” is a misnomer [47].

**References**

1. Constenla Umaña A. Estado actual de la subclasificación de las lenguas chibchenses y de la reconstrucción fonológica y gramatical del protochibchense. Estudios de lingüística Chibcha. 2008;27:117-135.

2. Constenla Umaña A. Chibchan languages. In: Campbell L, Grondona V, editors. The Indigenous languages of South America: a comprehensive guide. Berlin: De Gruyter; 2012. pp. 391-439.

3. O'Connor L, Muysken P. The Native languages of South America: origins, development, typology.Cambridge: Cambridge University Press; 2014.

4. Barrantes R, Smouse PE, Mohrenweiser HW, Gershowitz H, Azofeifa J, Arias TD, et al. Microevolution in lower Central America: genetic characterization of the Chibcha-speaking groups of Costa Rica and Panama, and a consensus taxonomy based on genetic and linguistic affinity. Am J Hum Genet. 1990;46:63-84.

5. Ruiz-Narváez EA, Santos FR, Carvalho-Silva DR, Azofeifa J, Barrantes R, Pena SD. Genetic variation of the Y chromosome in Chibcha-speaking Amerindians of Costa Rica and Panama. Hum Biol. 2005;77:71-91.

6. Bieber FR. Turning base hits into earned runs: improving the effectiveness of forensic DNA data bank programs. J Law Med Ethics. 2006;34:222-233.

7. Segura-Wang M, Barrantes R. Dermatoglyphic traits of six Chibcha-speaking Amerindians of Costa Rica, and an assessment of the genetic affinities among populations. Rev Biol Trop. 2009;57:357-369.

8. Melton PE, Briceño I, Gómez A, Devor EJ, Bernal JE, Crawford MH. Biological relationship between Central and South American Chibchan speaking populations: evidence from mtDNA. Am J Phys Anthropol. 2007;13:753-770.

9. Usme-Romero S, Alonso M, Hernandez-Cuervo H, Yunis EJ, Yunis JJ. Genetic differences between Chibcha and Non-Chibcha speaking tribes based on mitochondrial DNA (mtDNA) haplogroups from 21 Amerindian tribes from Colombia. Genet Mol Biol. 2013;36:149-157.

10. Hoopes JW, Fonseca Zamora O. Goldwork and Chibchan identity: endogenous change and diffuse unity in the Isthmo-Colombian Area. In: Quilter J, Hoopes JM, editors. Gold and power in ancient Costa Rica, Panama and Colombia. Washington DC: Dumbarton Oaks; 2003. pp. 49-89.

11. Roewer L, Nothnagel M, Gusmão L, Gomes V, González M, Corach D, et al. Continent-wide decoupling of Y-chromosomal genetic variation from language and geography in Native South Americans. PLoS Genet. 2013;9:e1003460.

12. Hoopes JW. The emergence of social complexity in the Chibchan world of Southern Central America and Northern Colombia, AD 300-600. J Archaeol Res. 2005;13:1-47.

13. Quilter J. Cobble circles and standing stones: archaeology at the Rivas Site, Costa Rica: University of Iowa Press; 2004.

14. Linares OF, Sheets PD, Rosenthal EJ. Prehistoric agriculture in tropical highlands. Science. 1975;187:17.

15. Mayo J, Mayo C, Karas V. La escultura precolombina del Área Intermedia. Aproximación al estudio estilístico, iconográfico y espacial del grupo escultórico de El Caño. In: Tisoc EM, Ciriaco RS, Licón EG editors. Producción de bienes de prestigio ornamentales y votivos de la América antigua. Deale: Syllaba Press; 2010. pp.99-111.

16. Fernández P, Qunitanilla I. Metallurgy, balls, and stone statuary in the Diquís delta, Costa Rica: Local production and power symbols. In: Quilter J, Hoopes JM, editors. Gold and power in ancient Costa Rica, Panama and Colombia. Washington, DC: Dumbarton Oaks; 2003. pp. 205-244.

17. Cooke R. Prehistory of Native Americans on the Central American land bridge: colonization, dispersal, and divergence. J Archaeol Res. 2005;13:129-187.

18. Cooke RG, Isaza II, Griggs J, Desjardins B, Sánchez LA. Who crafted, exchanged and displayed gold in pre-Columbian Panama. In: Quilter J, Hoopes JM, editors. Gold and power in ancient Costa Rica, Panama and Colombia. Washington DC: Dumbarton Oaks; 2003. pp. 91-158.

19. Martín J, Bustamante F, Holst I, Lara-Kraudy A, Redwood S, Sánchez-Herrrera L, et al. Ocupaciones prehispánicas en Isla Pedro González, Archipiélago de Las Perlas, Panamá. Aproximación a una cronología con comentarios sobre las conexiones externas. Lat Am Antiq. In Press.

20. Lothrop SK. Coclé: an archaeological study of Central Panama. Boston: Peabody Museum of Archaeology and Ethnology, Harvard University; 1942.

21. Lothrop SK. Metals from the Cenote of Sacrifice, Chichén-Itzá, Yucatán. Boston: Peabody Museum of Archaeology and Ethnology, Harvard University;1952;(10).

22. Bray WM. Sitio Conte metalwork in its pan-American context. In: Hearne P, Sharer RJ, editors. River of Gold: Precolumbian treasures from Sitio Conte. Philadelphia: Museum of Archaeology and Anthropology, University of Pennsylvania; 1992. pp. 33-46.

23. Cooke R. Cupica (Chocó): A reassessment of Gerardo Reichel-Dolmatoff’s fieldwork in a poorly studied region of the American tropics. In: Oyuela-Caycedo A, Scott Raymond J, editors. Recent Advances in the Archaeology of the Northern Andes. 39. Los Angeles: UCLA Institute of Archaeology; 1998. pp. 91-106.

24. Iizuka F, Cooke R, Frame L, Vandiver P. Inferring provenance, manufacturing technique, and firing temperatures of the Monagrillo ware (3520-1300 cal BC), Panama’s first pottery. In: Martinón-Torres M, editor. Craft and science: international perspectives on archaeological ceramics. Doha, Qatar: Bloomsbury Qatar Foundation; 2014. pp. 19-29.

25. Sauer CM. The early Spanish Main. Berkeley: University of California Press; 1964.

26. Castillero Calvo A. Conquista, evangelización y resistencia: triunfo o fracaso de la política indigenista? Panamá: Instituto Nacional de Cultura; 1995.

27. McLeod M. Spanish Central America: a socioeconomic history, 1520-1720. Austin: University of Texas Press; 2007.

28. Cooke RG, Sánchez LA, Carvajal D, Griggs JD, Isaza II. Transformaciones sociales y culturales de los amerindios de Panamá durante el siglo XVI: una perspectiva arqueológica y paleoecológica. Mesoamérica. 2003;45:1-34.

29. Ibarra Rojas E. Entre el dominio y la resistencia. Los pueblos indígenas del Pacífico de Nicaragua y Nicoya en el siglo XVI. San José: Editorial UCR; 2014.

30. Castillero Calvo A. Los metales preciosos y la primera globalización. Panamá: Editora Novo Art; 2008.

31. Quilter J, Hoopes JW. Gold and Power in Ancient Costa Rica, Panama, and Colombia. Washington DC: Dumbarton Oaks; 2003.

32. Ibarra Rojas E. Pueblos que capturan: esclavitud indígena al sur de América central del siglo XVI al XIX. San José: Editorial UCR; 2012.

33. Romoli K. Los de la lengua Cueva: los grupos indígenas del Istmo Oriental en la época de la Conquista Española. Santa Fé de Bogotá: Instituto Colombiano de Antropología e Instituto Colombiano de Cultura; 1987.

34. Vargas Sarmiento P. Los Emberá y los Cuna: impacto y reacción ante la ocupación española, siglos XVI y XVII EC. Bogotá: Instituto Colombiano de Antropología; 1993.

35. Arias TD. Una visión sintética del origen de los emberá y los waunaan en Colombia. Revista cultural lotería. 2003; 446: 53-64.

36. Loewen JA. Choco 1: Introduction and bibliography. Int J Am Linguist. 1963;29:239-263.

37. Arias TD. Los ‘cholos de Coclé’: origen, filogenia y antepasados indígenas, ¿los coclé o los ngöbé? Un estudio genético-histórico. Societas, Revista de ciencias sociales y humanísticas3. 2001; 3:55-88.

38. Jopling CF. Indios y negros en Panamá en los siglos XVI y XVII: Selecciones de los documentos del Archivo General de Indias. Guatemala: Centro de Investigaciones Regionales de Mesoamérica; 1994.

39. Perego UA, Lancioni H, Tribaldos M, Angerhofer N, Ekins JE, Olivieri A, et al. Decrypting the mitochondrial gene pool of modern Panamanians. PLoS One. 2012;7:e38337.

40. Martínez Mauri M. La autonomía indígena en Panamá: la experiencia del pueblo kuna (siglos XVI-XXI). Quito, Ecuador: Editorial Universitaria Abya Yala; 2011.

41. Arias T, Inaba T, Cooke R, Jorge L. A preliminary note on the transient polymorphic oxidation of sparteine in the Ngawbé Guaymí Amerindians: a case of genetic divergence with tentative phylogenetic time frame for the pathway. Clin Pharmacol Ther. 1988;44:343-352.

42. Young PD. Notes on the ethnohistorical evidence for structural continuity in Guaymí society. Ethnohistory. 1970;17:11-29.

43. Young PD. Ngawbe: Tradition and change among the Western Guaymi of Panama. 1971.

44. Gunn R, Gunn M. Fonología Bokotá. Patrimonio Histórico (Panama). 1984;1:69-91.

45. Howe J. A people who would not kneel: Panama, the United States, and the San Blas Kuna: Smithsonian Inst Press; 1998.

46. Helms M. Ancient Panama: Chiefs in Search of Power. Austin: University of Texas. 1979.

47. Cooke R. Review of Ancient Panama: Chiefs in Search of Power. Ethnohistory. 1984; 31:115-116.
